# On the Origin and Treatment of Purulent Ophthalmia

**Published:** 1876-05

**Authors:** 


### ON THE ORIGIN AND TREATMENT OF PURULENT OPHTHALMIA.

In the *Wiener Medizinische Zeitung*, May 25, Professor Arlt brings to end a series of papers upon purulent ophthalmia, and in a short *resume* thus sums up the conculsions at which he had arrived. Although purulent ophthalmia very commonly appears amongst the members of communities, yet it not unfrequently attacks the eyes of individuals amongst the poorer and lower classes, who are already suffering from any of the milder forms of ophthalmia; it matters little what the nature of this may be, whether it be granular or trachomatous, or any other variety of inflammation; it may appear after a short interval, and it may lead to a rapid destruction of the eyesight by invading the cornea, or to its slow and gradual extinction by causing trichiasis and entropium. In many cases, minute grayish bodies may be seen on the under surface of the upper lid, and especially in the mucous membrane above the lid. The ophthalmia is generally acute, if it be caused by inoculation from the genital organs, but it is usually chronic when it spreads from eye to eye, or when it is brought about by exposure to draught. The activity of the contagious matter depends much upon the concentration of the material, and upon the length of time during which the conjunctiva is exposed to its influence; its power is favored by heat and retarded by cold. The occurrence of one single case within a community may lead to the appearance of an epidemic, the contagion being conveyed as much by the air as by all kinds of instruments and utensils, more especially when the weather is warm and moist. It appears that the disease may have its origin in atmospheric influences alone, such, for instance, as sudden changes of the weather, and if the air be charged with dust or smoke. According to Professor Arlt, it is not certain that purulent ophthalmia is more rife amongst military than in civil communities, nor does it appear to him conclusively shown that the disease had its origin in Egypt, inasmnch as Egypt was esteemed by the Greeks and Romans as a very healthy country, and the assertion that Cyrus applied to Egypt for the aid of physicians does not prove more than that the Egyptians in those days were a cultivated people, and were in a position to afford the assistance which was asked. There is no reason to suppose that there is any change of structure in the eyes of scrofulous subjects which should render them especially liable to the disease.

In conclusion, as regards treatment, Professor Arlt is quite at a loss how to suggest any safeguards against the disease; for, although we look upon it as contagious, we cannot isolate all or any who have been exposed to it, and it very frequently happens that those who have been exposed are utterly unaware of the fact, and but too often unwilling to submit to any treatment until the disease is far advanced, and, according to our present knowledge, it would be quite impossible for medical men to examine the eyes of all those under their care, and who may in any way have exposed themselves, or whose eyes may at any time become attacked.—*London Medical Record*, August 16, 1875.

